# Assessing the impact of routine prenatal ultrasound on pregnancy and perinatal outcomes in low-resource settings: A systematic review protocol

**DOI:** 10.1016/j.mex.2026.104046

**Published:** 2026-07-13

**Authors:** Yusif Yakub, Ritu Mogra, Naomi Noguchi, Mohammed Amin, Hala Phipps, Bradley de Vries

**Affiliations:** a Central Clinical School, Faculty of Medicine and Health, The University of Sydney, Science Rd., Camperdown, NSW, 2050, Australia; b Sydney School of Public Health, Faculty of Medicine and Health, The University of Sydney, Science Rd., Camperdown, NSW, 2050, Australia; c School of Nursing and Midwifery, Faculty of Health, Deakin University, 221 Burwood Highway, Burwood, VIC, 3125, Australia; d Susan Wakil School of Nursing and Midwifery, Faculty of Medicine and Health, The University of Sydney, Science Rd., Camperdown, NSW, 2050, Australia; e Sydney Institute for Women, Children, and their Families, Sydney Local Health District, Sydney, Australia; f Reproduction and Perinatal Centre, Faculty of Medicine and Health, The University of Sydney, Sydney, Australia

**Keywords:** Ultrasound, Prenatal, Maternal mortality, Low-resourced setting, Pregnancy

## Abstract

**Background:**

Obstetric ultrasound is integral to pregnancy care, yet access remains limited in low-resource settings, potentially contributing to preventable maternal and perinatal mortality.

**Objective:**

To systematically identify, critically appraise and synthesise evidence on the impact of routine prenatal ultrasound on pregnancy, maternal and perinatal outcomes in low-resource settings.

**Eligibility criteria:**

Pregnant women in low-income countries (World Bank GNI per capita ≤US$1135) or low-resource facilities in lower-middle-income countries. Eligible studies compare routine prenatal ultrasound against any setting where ultrasound is not systematically offered to all pregnant women, including no ultrasound access, selective ultrasound only, or ultrasound available but not routinely provided. All comparator types will be included in the primary pooled analysis, with comparator type explored as a pre-specified subgroup.

**Information sources:**

Seven databases (MEDLINE, Embase, CINAHL, Cochrane CENTRAL, Scopus, Maternity and Infant Care, Global Index Medicus) searched from January 1980 to 30 June 2026, with no language restriction.

**Risk of bias:**

The Scottish Intercollegiate Guidelines Network (SIGN) checklist for randomised trials; Newcastle-Ottawa Scale (NOS) for observational studies.

**Synthesis:**

Random-effects meta-analysis (DerSimonian-Laird); narrative synthesis following SWiM where pooling is not feasible.

**Certainty of evidence:**

GRADE applied across five domains for co-primary outcomes (perinatal mortality; maternal mortality) and all secondary outcomes.

**Registration:**

PROSPERO CRD42023474088.

## Graphical abstract

**Figure:**
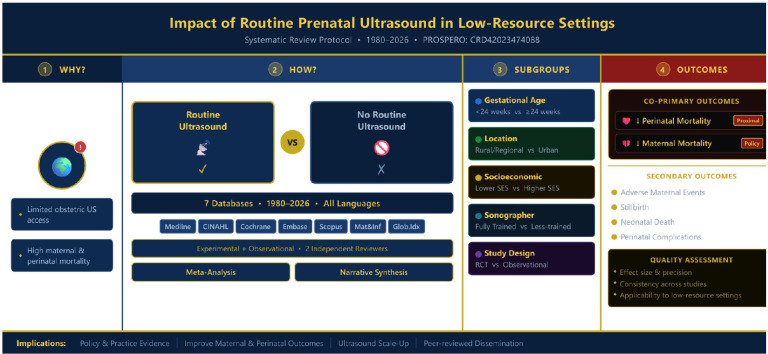

**Specifications table**

**Table:** 

**Subject area**	Medicine and Dentistry
**More specific subject area**	Obstetrics and Gynaecology
**Name of your protocol**	Assessing the Impact of Routine Prenatal Ultrasound on Pregnancy and Perinatal Outcomes in Low-Resource Settings: A Systematic Review Protocol
**Reagents/tools**	‘Non-Applicable’
**Experimental design**	This systematic review protocol evaluates the impact of routine prenatal ultrasound on pregnancy, maternal and perinatal outcomes in low-resource settings. Seven databases will be searched for experimental and observational studies published between January 1980 and 30 June 2026 with no language restriction. The review adopts co-primary outcomes of perinatal mortality (stillbirth plus neonatal death) and maternal mortality. Risk of bias will be assessed using the Scottish Intercollegiate Guidelines Network (SIGN) checklist for randomised trials and the Newcastle-Ottawa Scale (NOS) for observational studies, with domain-level judgements informing certainty of evidence ratings using GRADE. Pre-specified subgroup analyses will explore gestational age at ultrasound (primary cutoff <24 weeks, aligned with WHO recommendations), location, socioeconomic status, and sonographer training level.
**Trial registration**	PROSPERO: CRD42023474088
**Ethics**	Ethics approval is not required for this systematic review, as it entails the analysis of data from previously conducted published studies.
**Value of the Protocol**	Addressing a critical gap in evidence: Despite the widespread use of obstetric ultrasound in pregnancy care, there is limited synthesized evidence on its impact specifically within low-resource settings, where maternal and perinatal mortality rates remain disproportionately high. Informing policy and practice: Findings from this systematic review have the potential to guide healthcare policies and resource allocation decisions, supporting efforts to improve access to routine prenatal ultrasound in underserved regions. Advancing global maternal health: By examining key factors such as gestational timing, location, and sonographer training, this review can contribute to targeted strategies that reduce maternal and perinatal complications in low-resource settings worldwide.

## Background

The impact of obstetric ultrasound in enhancing outcomes for mothers and their newborns is a growing area of focus and an important element in global antenatal care [1,2]. Access to ultrasound services may play a crucial role in reducing maternal mortality rates and improving neonatal health outcomes, particularly in resource-limited settings [3].

The main goals of a first trimester routine ultrasound are to confirm intrauterine pregnancy and establish gestational age [4]. It also helps detect any abnormalities in the uterus or ovaries and checks for chorionicity and amnionicity in multiple gestations [5]. Routine ultrasounds in the second and third trimesters confirm the number of fetuses and placenta location [6], as well as identify any fetal abnormalities [7]. These ultrasounds also help to detect placental anomalies, which significantly affects fetal and perinatal outcomes [8].

Obstructed labour, unsafe abortion, and haemorrhage collectively constitute a significant portion of maternal fatalities [9], and ultrasound emerges as a promising strategy for effectively managing these risks [10,11]. The WHO's global guidelines for pregnancy care in 2016 and 2022 incorporated a recommendation for at least one obstetric ultrasound scan before 24 weeks of gestation [12,13].

An estimated 99% of maternal deaths occur in low-resource countries [14], with sub-Saharan Africa bearing the weight of approximately two-thirds of the world's maternal mortality [15]. The region also experiences the highest rates of newborn mortality [16]. In much of the sub-Saharan African region, only 6% of pregnant women in rural areas are estimated to have access to obstetric ultrasound, while those in urban settings have a slightly higher coverage of around 30% [17]. Lack of access of antenatal ultrasound in sub-Saharan Africa is a significant challenge in addressing the enormous burden of perinatal morbidity and mortality [17].

The objective of this systematic review is to address how the use of routine prenatal ultrasound impact on pregnancy and perinatal outcomes for women giving birth in low-resource settings and their babies, compared with no ultrasound or only using ultrasound when specifically indicated.

## Description of protocol

### Methods

#### Protocol design and registration

This systematic review is not an update of a previous systematic review, and the protocol has been prepared consistent with the Preferred Reporting Items for Systematic Reviews and Meta-Analysis Protocols 2015 checklist [18].

Our systematic review protocol is registered with the International Prospective Register of Systematic Reviews (http://www. crd.york.ac.uk/PROSPERO) on 29 October 2023 with registration number CRD42023474088.

### Eligibility criteria

#### Study designs

The review will include studies that compare routine ultrasound utilization in antenatal care with no routine ultrasound utilization in antenatal care. It will include randomised controlled trials with randomisation at the individual and cluster level. The review will also include prospective and retrospective comparative observational studies including cohort studies and case-controlled studies. Studies that reported routine prenatal ultrasound utilization in low-resource settings will be included. Non-comparative studies and studies that do not assess ultrasound in pregnancy will be excluded. The screening process will exclude commentaries, letters, and editorials.

### Participants and settings

Eligible participants will be pregnant women receiving antenatal care in low-resource settings. For the purposes of this review, 'low-resource settings' are defined using a two-tier operational framework. The primary definition encompasses countries classified as low-income by the World Bank based on a gross national income (GNI) per capita at or below US$1135 (World Bank FY2026 classification), reflecting severe and systemic economic constraints on healthcare provision. The secondary definition extends to subnational regions or health facilities within lower-middle-income countries (GNI per capita US$1136–US$4495) where access to obstetric ultrasound is explicitly documented as limited or absent by study authors [19]. This broadened but operationally bounded definition captures the clinical reality in settings such as rural sub-Saharan Africa and South Asia, where profound urban-rural disparities in imaging access persist even within countries that exceed the low-income threshold. Studies set in upper-middle-income or high-income countries will be excluded unless conducted in facilities or communities explicitly characterised as resource-limited for antenatal imaging.

Intervention and Comparison Groups

The intervention is the provision of routine antenatal ultrasound, defined as at least one ultrasound examination offered systematically to all pregnant women as part of standard antenatal care, regardless of clinical indication. This includes single-scan programmes (typically one scan offered at any gestational age) and multi-scan programmes (two or more scans at defined gestational windows).

The comparator is any setting in which routine antenatal ultrasound is not systematically offered to all pregnant women. This encompasses: (a) no ultrasound access; settings in which obstetric ultrasound equipment is absent or non-functional, (b) selective or indicated ultrasound only; ultrasound performed solely in response to a clinical indication, and (c) ultrasound available but not systematically offered; equipment exists but is not integrated into routine antenatal care protocols. All three comparator types will be included in the primary pooled analysis. Comparator type will be explored as a pre-specified subgroup analysis if substantial heterogeneity is observed (I-squared >60%), given that the counterfactual experience of women in each category may differ. Studies in which the comparator type is not clearly described will be included but flagged.

### Other eligibility criteria

Studies will be included if perinatal and maternal mortality or any of the outcomes listed in the additional outcomes section below are reported. Publications from 1st January 1980 to 30th June 2026 reported in any language will be included. Studies in other languages apart from English will be initially translated using Google Translate, and key data will be confirmed by bilingual colleagues fluent in the respective languages.

### Information sources

We will search Medline (OvidSP), CINAHL, Cochrane Library/CENTRAL, Embase (OvidSP), Scopus, Maternity and Infant Care, and Global Index Medicus. To thoroughly cover the existing literature, we will examine the reference lists of the studies included and pertinent reviews that have been identified through our search for other relevant papers for possible inclusion.

### Search strategy

Searches will include terms such as ultrasound, ultrasonography, sonography, scan, imaging, maternal, prenatal, antenatal, pregnancy outcomes, perinatal outcomes, sub-Saharan Africa and other related terms. The search strategy which is prospectively designed has been formally peer-reviewed by the University of Sydney Faculty of Health designated librarian prior to execution. The planned search strategies are below:

### Search strategy for Ovid MEDLINE(R) ALL <1980 to June 30, 2026

1. ((ultraso* or sonograph* or scan* or imag*) adj3 (pregnan* or gestation or cyesis or antenatal or prenatal or obstetric*)).mp or Ultrasonography, Prenatal/ or

"antenatal care".mp or "pregnancy outcome*".mp or "perinatal outcome*".mp

2. developing countries/ or exp Africa South of the Sahara/ or exp Africa, Western/ or exp Africa, Eastern/ or exp Africa, Central/ or exp Asia, Southern/

3. (developing countr* or low* income countr* or low* resource countr* or low* middle income countr* or LMIC or Ghana or Ethiopia or Afghanistan or Benin or Rwanda or

Zimbabwe or Niger or Somalia or South Sudan or Sierra Leone or Tanzania or Togo or

Uganda or Zambia or Guinea or Guinea-Bissau or Haiti or Democratic People's Republic of Korea or North Korea or Liberia or Madagascar or Malawi or Mali or Mozambique or

Eritrea or Democratic Republic of Congo or Burundi or Burkina Faso or Chad or

Comoros or Central African Republic).mp

4. 2 or 3

5. 1 and 4

### The search technique/strategy for Cochrane Library/CENTRAL database will be as follows

1. ((ultraso* or sonograph* or scan* or imag*) adj3 (pregnan* or gestation or cyesis or antenatal or prenatal or obstetric*)).mp or Ultrasonography, Prenatal/ or

"antenatal care".mp or "pregnancy outcome*".mp or "perinatal outcome*".mp

2. developing countries/ or (mh "Africa South of the Sahara") or (mh "Asia, Southern")

3. (developing countr* or low* income countr* or low* resource countr* or low* middle income countr* or LMIC or Ghana or Ethiopia or Afghanistan or Benin or Rwanda or

Zimbabwe or Niger or Somalia or South Sudan or Sierra Leone or Tanzania or Togo or

Uganda or Zambia or Guinea or Guinea-Bissau or Haiti or Democratic People's Republic of Korea or North Korea or Liberia or Madagascar or Malawi or Mali or Mozambique or

Eritrea or Democratic Republic of Congo or Burundi or Burkina Faso or Chad or

Comoros or Central African Republic).mp

4. 2 or 3

5. 1 and 4

### Search strategy for Global Index Medicus

(((ultraso* OR sonograph* OR scan* OR imag*) AND (obstetric* OR antenatal OR prenatal

OR pregnancy OR gestation OR cyesis OR "antenatal care" OR "pregnancy outcome*"

OR "perinatal outcome*")))

AND

("low middle income countr*" OR Ghana OR Ethiopia OR Afghanistan OR Benin OR Rwanda

OR Zimbabwe OR Niger OR Somalia OR "South Sudan" OR "Sierra Leone" OR Tanzania OR

Togo OR Uganda OR Zambia OR Guinea OR "Guinea-Bissau" OR Haiti OR

"Democratic People's Republic of Korea" OR "North Korea" OR "Liberia" OR

Madagascar OR Malawi OR Mali OR Mozambique OR Eritrea OR

"Democratic Republic of Congo" OR Burundi OR "Burkina Faso" OR Chad OR

Comoros OR "Central African Republic")

### Search strategy for Embase (Ovid)

1. ((ultraso* or sonograph* or scan* or imag*) adj3 (pregnan* or gestation or cyesis or antenatal or prenatal or obstetric*)).mp or fetus echography/ or

"antenatal care".mp or "pregnancy outcome*".mp or "perinatal outcome*".mp

2. developing countries/ or exp western Africa/ or exp eastern Africa/ or exp sub-Saharan Africa/ or exp South Asia/

3. (developing countr* or low* income countr* or low* resource countr* or low* middle income countr* or LMIC or Ghana or Ethiopia or Afghanistan or Benin or Rwanda or

Zimbabwe or Niger or Somalia or South Sudan or Sierra Leone or Tanzania or Togo or

Uganda or Zambia or Guinea or Guinea-Bissau or Haiti or Democratic People's Republic of Korea or North Korea or Liberia or Madagascar or Malawi or Mali or Mozambique or

Eritrea or Democratic Republic of Congo or Burundi or Burkina Faso or Chad or

Comoros or Central African Republic).mp

4. 2 or 3

5. 1 and 4

### Search strategy for CINAHL via EBSCO

1. (ultraso* OR sonograph* OR scan* OR imag*) N3 (pregnan* OR gestation OR cyesis OR antenatal OR prenatal OR obstetric*) OR MH "Ultrasonography, Prenatal+" OR

"antenatal care" OR "pregnancy outcome*" OR "perinatal outcome*"

2. MH "Africa South of the Sahara+" OR MH "Asia+" OR "low-resource settings" OR

"resource-limited settings" OR "developing countries" OR LMICs OR Ghana OR

Ethiopia OR Afghanistan OR Benin OR Rwanda OR Zimbabwe OR Niger OR Somalia OR

"South Sudan" OR "Sierra Leone" OR Tanzania OR Togo OR Uganda OR Zambia OR

Guinea OR "Guinea-Bissau" OR Haiti OR "Democratic People's Republic of Korea" OR

"North Korea" OR Liberia OR Madagascar OR Malawi OR Mali OR Mozambique OR

Eritrea OR "Democratic Republic of Congo" OR Burundi OR "Burkina Faso" OR

Chad OR Comoros OR "Central African Republic"

3. 1 AND 2

### SCOPUS search strategy

(ultraso* OR sonograph* OR scan* OR imag*) W/3 (obstetric* OR antenatal OR prenatal

OR pregnan* OR gestation OR cyesis OR "antenatal care" OR "pregnancy outcome*" OR

"perinatal outcome*")

AND

("low middle income countr*" OR Ghana OR Ethiopia OR Afghanistan OR Benin OR Rwanda

OR Zimbabwe OR Niger OR Somalia OR "South Sudan" OR "Sierra Leone" OR Tanzania OR

Togo OR Uganda OR Zambia OR Guinea OR "Guinea-Bissau" OR Haiti OR

"Democratic People's Republic of Korea" OR "North Korea" OR "Liberia" OR

Madagascar OR Malawi OR Mali OR Mozambique OR Eritrea OR

"Democratic Republic of Congo" OR Burundi OR "Burkina Faso" OR Chad OR

Comoros OR "Central African Republic")

### Maternity and Infant Care search strategy

1. (ultraso* or sonograph* or scan* or imag*) adj3 (pregnan* or gestation or cyesis or antenatal or prenatal or obstetric*) or "antenatal care" or

"pregnancy outcome*" or "perinatal outcome*"

2. (developing countr* or low* income countr* or low* resource countr* or low* middle income countr* or LMIC or Ghana or Ethiopia or Afghanistan or Benin or Rwanda or

Zimbabwe or Niger or Somalia or South Sudan or Sierra Leone or Tanzania or Togo or

Uganda or Zambia or Guinea or Guinea-Bissau or Haiti or Democratic People's Republic of Korea or North Korea or Liberia or Madagascar or Malawi or Mali or Mozambique or

Eritrea or Democratic Republic of Congo or Burundi or Burkina Faso or Chad or

Comoros or Central African Republic)

3. 1 AND 2

### Study records

#### Data management

The search outcomes will be transferred into Covidence systematic review management software for citation screening, deduplication, and full-text review. Any duplicates not automatically identified by Covidence will be removed manually.

#### Study selection

One investigator (YY) will conduct an initial search after which all duplicates will be removed. Then, two investigators (YY and MA) will screen article titles and abstracts to exclude studies that do not meet the predetermined eligibility criteria. The full text of all potentially eligible papers will be examined independently by two investigators (YY and MA) for inclusion. Disagreements will be resolved by consensus among a panel of four investigators (RM, NN, HP and BdV). Reference lists of selected articles and pertinent reviews will be examined by two investigators (YY and MA) for other relevant papers for possible inclusion.

#### Data collection process

Two authors (YY and MA) will independently extract relevant information using a piloted data collection form. The extracted data will encompass demographic details, study type, study setting, intervention specifics, and all documented patient-relevant outcomes.

Disagreement among reviewers will be addressed through discussions involving four arbitrators (RM, NN, HP, BdV). Study authors will be contacted to resolve any uncertainties

#### Data items

We will extract the intervention, comparator, participant characteristics (such as pregnant women in low-resource settings), outcomes listed under ‘additional outcomes’ below, number of routine ultrasounds, timing of routine ultrasounds, country(s), availability of ultrasounds to women in the comparison group, mortality rate across maternal age, the geographic distance from ultrasound care and socio-economic status of the mothers.

### Outcomes and prioritization

This review adopts two co-primary outcomes reflecting two key clinical pathways through which routine prenatal ultrasound may reduce harm in low-resource settings. The co-primary outcomes are: [1] Perinatal mortality, defined as stillbirth at or beyond 22 weeks of gestation plus neonatal death within 28 days of birth in an infant weighing at least 500 g or, where birthweight is unknown, born at or beyond 22 weeks' gestation. Perinatal mortality could be improved through s earlier detection of malpresentation, placenta praevia, multiple gestation, fetal growth restriction, and fetal anomalies, each of which, when acted upon, may prevent perinatal death [2] Maternal mortality, defined as the death of a woman while pregnant or within 42 days of termination of pregnancy, irrespective of the duration and site of the pregnancy, from any cause related to or aggravated by the pregnancy or its management (ICD-10 definition) [20].

Maternal mortality is retained as a co-primary outcome given its paramount policy relevance in low-resource settings, where 99% of global maternal deaths occur, and because ultrasound contributes to reducing leading causes including obstructed labour, placenta praevia-related haemorrhage, and complications of multiple gestation. This outcome will be reported with appropriate caution given the anticipated rarity of events and limited reporting in primary studies. For both co-primary outcomes, evidence will be assessed using GRADE, and findings will be interpreted in the context of clinical plausibility and the causal logic described in the conceptual framework below. The two co-primary outcomes will not be combined into a composite endpoint.

### Additional outcome(s)

Other outcomes include:

**Stillbirth:** Defined as a lifeless infant weighing at least 500 g at birth or, if weight is unknown, born after at least 22 weeks' gestation [21,22]. Although stillbirth contributes to the composite perinatal mortality co-primary outcome, it is retained here as a secondary outcome to enable separate reporting, given that stillbirth and neonatal death could be impacted through distinct causal pathways from prenatal ultrasound and require individual GRADE certainty assessments.

**Neonatal death:** Defined as death occurring within 28 days of birth in an infant whose birth weight was at least 500 g or, if weight is unknown, born after at least 22 weeks' gestation [21]. Although neonatal death contributes to the composite perinatal mortality co-primary outcome, it is retained here as a secondary outcome to enable separate reporting, given that it may reflect a distinct causal pathway, particularly relating to detection of fetal anomalies, preterm birth management, and resuscitation preparedness facilitated by antenatal ultrasound, and requires an individual GRADE certainty assessment.

**Post-partum haemorrhage:** This is excessive bleeding after childbirth.

**Antepartum haemorrhage:** This is genital bleeding during pregnancy after the 24th week of pregnancy up to delivery.

**Maternal blood transfusion:** This is the treatment with intravenous blood products to restore haemoglobin level.

**Caesarean section:** This is a surgical procedure by which one or more babies are delivered through an incision in the mother’s abdomen.

**Obstructed labour (as defined by the authors):** This is when the baby is not exiting the pelvis because it is physically blocked during childbirth although the uterus contracts normally.

**Maternal admission to ICU:** Maternal admission to the adult intensive care unit.

**Low 5-minute Apgar score (< 7 or < 4):** Apgar score < 7 will be reported if available. Apgar score < 4 will be reported if available. If the paper does not report on a cut-off for low Apgar score or an alternative cut-off is reported, then results may be pooled using the author’s definition.

**Abnormal umbilical arterial cord gases (as defined by the authors):** Authors’ definitions will be used.

**Gestational age at birth:** This is the measure of the age of a pregnancy taken from the beginning of the woman’s last menstrual period, or the corresponding age of the pregnancy as estimated by a more accurate method, if available.

**Preterm birth < 37 weeks:** This is a birth that occurs before the 37th week of pregnancy.

### Risk of bias in individual studies

The Scottish Intercollegiate Guidelines Network (SIGN) [23] checklist will be used to critically appraise randomised controlled trials, assessing domains including selection bias, randomisation and concealment bias, baseline comparability, blinding, attrition, and generalisability. The Newcastle-Ottawa Scale (NOS) [24] will be used for observational studies to assess selection bias, comparability bias, and outcome bias. These tools cover the key risk-of-bias domains relevant to this review.

It is important to note that any risk-of-bias assessment tool that comprehensively covers all important risk domains is appropriate for use in a systematic review. While RoB 2 and ROBINS-I represent the current Cochrane-preferred standard, their proper operationalisation demands advanced epidemiological training: RoB 2 requires users to determine whether they are estimating the effect of intervention assignment or adherence before responding to all subsequent questions, a high-level conceptual distinction that is difficult to operationalise in practice; ROBINS-I requires a sound understanding of causal inference and the target trial framework. Given the composition of our review team, SIGN and NOS provide a rigorous, validated, and practically appropriate framework. Domain-level risk-of-bias judgements from both tools will directly inform GRADE certainty ratings.

### Data synthesis

Where meta-analysis is feasible, a random-effects model (DerSimonian-Laird method) will be used, given the anticipated heterogeneity in settings, scan timing, operator training, and study design. Dichotomous outcomes will be pooled as Mantel-Haenszel risk ratios (RR) with 95% confidence intervals; continuous outcomes as mean differences (MD) or standardised mean differences (SMD).

Adjusted effect estimates will be prioritised over unadjusted estimates where both are reported. Statistical heterogeneity will be assessed using the I-squared statistic, Cochran's Q test, and tau-squared, with prediction intervals reported for all pooled estimates. For sparse outcomes including maternal mortality, the Peto odds ratio method will be applied, with continuity correction where zero events occur. Cluster-randomised trials will be included where appropriate adjustment for clustering has been undertaken. Where adjustment for clustering has not been undertaken, a design-effect correction will be used based on the intracluster correlation coefficient.

Where pooling is not feasible due to clinical or methodological heterogeneity, narrative synthesis will follow the Synthesis Without Meta-Analysis (SWiM) reporting framework. Sensitivity analyses will be conducted restricted to studies at low risk of bias, RCTs only, studies using the primary comparator, and studies published from 2000 onwards. Analyses will be conducted in R (meta and metafor packages), with RevMan Web used for forest plot and risk-of-bias figure generation.

Pre-specified subgroup analyses will explore clinical and methodological sources of heterogeneity. The primary gestational age subgroup compares ultrasound performed at <24 weeks versus 24 or more weeks, consistent with WHO recommendations and ISUOG guidelines, as earlier scanning enables detection of structural anomalies, multiple gestation, placenta praevia, and more accurate gestational age dating.

Additional subgroups include location, socioeconomic status, and sonographer training level, each with pre-specified definition hierarchies to address cross-study variation.

Subgroup analyses will be performed for the following groups:

1. Gestational age at ultrasound: primary subgroup, <24 completed weeks versus 24 or more weeks (aligned with WHO and ISUOG recommendations).
2. Rural/regional location versus metropolitan
3. Higher socioeconomic status versus lowest socioeconomic status
4. Fully trained sonographers versus sonographers with less training
5. Randomised controlled trials versus observational studies

Rurality classifications will follow a pre-specified hierarchy to account for cross-study variation in definitions. Where studies use a national census-based or government-defined urban/rural classification, that definition will be applied and reported. Where no formal classification is used, the following operational definitions will be applied: metropolitan/urban- facilities located within or serving populations in urban centres with populations exceeding 100,000, with access to tertiary-level obstetric services; rural/regional- facilities located in areas with limited access to tertiary hospitals, defined as travel time of more than two hours or a catchment population of fewer than 50,000. Studies where the above options are not feasible, the author's definitions will be used.

Socioeconomic status will be compared as higher versus lower, dividing studies into two broad categories to retain all available data. Where income quintile or wealth index data are reported, studies in the upper half of the distribution will define higher SES and those in the lower half will define lower SES. Where authors use categorical definitions, those will be applied and reported. Where only proxy measures are available (educational attainment, household assets, DHS wealth quintiles), these will be used with the measure clearly described.

Sonographer training level will be categorised as follows, with a pre-specified mapping approach to handle cross-study variation. Fully trained sonographers are defined as individuals who have completed a certified diagnostic medical sonography programme accredited by a recognised professional body (example, Australasian Sonographer Accreditation Registry, American Registry for Diagnostic Medical Sonography (ARDMS), or national equivalent) with two or more years of supervised clinical experience in obstetric ultrasound.

Task-shifted or partially trained operators are defined as midwives, nurses, or doctors who have received structured short-term point-of-care ultrasound training of three months or less duration, or fewer than 100 supervised obstetric scans, without formal professional accreditation. Where studies describe training in different terms, the level will be mapped to the nearest category based on duration, supervision structure, and scope of practice, with the mapping decision documented. Where training level cannot be ascertained from study reporting, studies will be included in the main analysis but excluded from the training-level subgroup analysis. A narrative summary of training approaches across included studies will supplement the quantitative subgroup analysis, given the expected heterogeneity in training descriptions across low-resource settings globally.

### Meta-bias(es)

Funnel plots will be constructed to assess publication bias where ten or more studies contribute data to the meta-analyses.

### Confidence in cumulative evidence

The certainty of evidence will be assessed for each outcome using the Grading of Recommendations Assessment, Development and Evaluation (GRADE) [25] framework. All five domains will be evaluated: risk of bias, informed by SIGN and NOS domain-level assessments; inconsistency, based on I-squared, prediction intervals, and directional consistency of effects across studies; indirectness, considering whether study populations, ultrasound protocols, and outcomes are representative of the target low-resource settings; imprecision, assessed against optimal information size criteria and confidence interval width; and publication bias, evaluated using funnel plot asymmetry where ten or more studies contribute to a meta-analysis. Starting certainty will be rated as High for randomised trial evidence and Low for observational evidence. Upgrading will be considered where the effect is large (RR >2.0 or <0.5), a dose-response gradient is present, or all plausible residual confounders would be expected to attenuate the observed effect. GRADE evidence profiles and Summary of Findings tables will be generated for both co-primary outcomes and all secondary outcomes using GRADEpro GDT software.

### Protocol validation

The validity of this protocol rests on the rigour of its design rather than findings yet to be generated. A comprehensive, language-unrestricted search across seven databases ensures reproducible literature coverage. Eligibility criteria are explicitly defined using PICO principles, with risk-of-bias assessed using validated and widely accepted tools: the Scottish Intercollegiate Guidelines Network (SIGN) checklist for randomised controlled trials and the Newcastle-Ottawa Scale (NOS) for observational studies. Both tools comprehensively cover the key risk-of-bias domains relevant to this review. Domain-level judgements from both tools will directly inform GRADE certainty ratings. Subgroup analyses are pre-specified and operationally defined with explicit definition hierarchies to address cross-study variation, and GRADE will be applied across all five domains to transparently link conclusions to the certainty of the underlying evidence. Prospective registration with PROSPERO (CRD42023474088) and adherence to PRISMA-P 2015 guidelines provide independent verification of methodological integrity prior to conduct of the review.

## Limitations

Several anticipated limitations should be acknowledged. First, substantial intervention heterogeneity is expected, as 'routine prenatal ultrasound' varies widely in timing, number of scans, operator training, and equipment quality across included studies, which may limit the interpretability of pooled estimates. Second, many eligible studies are likely to be observational, introducing risks of confounding by indication, socioeconomic factors, and healthcare-seeking behaviour that cannot be fully adjusted for in analysis. Third, contamination between intervention and comparison groups is possible in settings where ultrasound is available sporadically, blurring the boundary between routine and selective use.

Fourth, maternal mortality, a key outcome, is infrequently and inconsistently reported in studies evaluating ultrasound programmes, which may limit statistical power for this specific endpoint. Fifth, variation in access to emergency obstetric care following identification of complications on ultrasound will differ substantially across settings, potentially modifying the effect of ultrasound on clinical outcomes in ways that are difficult to disaggregate. Sixth, publication bias may favour studies from better-resourced settings with more favourable outcomes, limiting the generalisability of findings to the most resource-constrained contexts. These limitations will be addressed through pre-specified sensitivity analyses, transparent reporting using GRADE, and narrative synthesis where quantitative pooling is not appropriate.

## Related research article

“None.”

## For a published article

“None.”

## CRediT authorship contribution statement

**Yusif Yakub:** Conceptualization, Methodology, Investigation, Writing – original draft, Project administration. **Ritu Mogra:** Methodology, Validation, Writing – review & editing. **Naomi Noguchi:** Methodology, Validation, Writing – review & editing. **Mohammed Amin:** Conceptualization, Methodology, Investigation. **Hala Phipps:** Validation, Writing – review & editing, Supervision. **Bradley de Vries:** Conceptualization, Methodology, Validation, Writing – review & editing, Supervision.

## Declaration of competing interest

The authors declare that they have no known competing financial interests or personal relationships that could have appeared to influence the work reported in this paper.

## Data Availability

No data was used for the research described in the article.
